# Work-related suicide: an international social justice perspective

**DOI:** 10.1007/s10611-025-10237-2

**Published:** 2025-11-11

**Authors:** Steven Bittle, Sarah Waters, China Mills, Loic Lerouge

**Affiliations:** 1 Department of Criminology, University of Ottawa, Ottawa, Canada; 2 School of Languages, Cultures and Societies, University of Leeds, Leeds, UK; 3 Head of Research Healing Justice London, London, UK; 4 Centre de droit comparé du travail et de la sécurité sociale Centre for Comparative Labour and Social Security Law Chaire internationale d’études comparées de la santé au travail (CIECST), COMPTRASEC UMR5114, CNRS- University of Bordeaux, Bordeaux, France

**Keywords:** Suicide, Work-related suicide, Social justice, Criminology of work

## Abstract

This paper explores what the study of work-related suicide can contribute to emerging discussions of a “criminology of work.” It does so by chronicling work-related suicide as an under-researched and largely unrecognized phenomenon that requires a social justice lens in which the focus is on *work*-related suicide, not work-related *suicide*. The vast majority of suicides today occur amongst working-age adults and work or working conditions are contributory factors in 10% to 13% of all suicides. Extending beyond a potentially narrow mental health perspective in which suicide is framed as the outcome of individualised psychology, a social justice framework emphasizes the need to confront the varied (social, economic, political, cultural, historical) conditions that make life unliveable for some workers. It is concerned with transforming the structural conditions of work in contemporary globalized economies, not just interventions that address the impact of work for individual workers. In this sense, the paper challenges those interested in a “criminology of work” to consider the harmful conditions of work as a collective social justice issue that reflects the rules, policies and laws, and underlying ideologies and values, that shape all our working lives.

## Introduction

The vast majority of suicides today occur amongst working-age adults and work or working conditions have been identified as contributory factors in 10% to 13% of all suicides (LaMontagne et al., 2024). Yet the nature of work, workplaces, workplace health and safety, or the links between work and suicide, are not typical criminological subjects. With few exceptions, such as the literature on sex work (e.g., Bruckert & Hannem, 2013), work has been of criminological concern mainly in the context of examining the link between unemployment and conventional crimes (Kapuscinski et al., 1998; Raphael & Winter-Ebmer 2001). There is also limited criminological research on unsafe workplaces (for exceptions, see Almond, 2010; Bittle, 2012; Tombs & Whyte, 2007), and even fewer studies on the links between work and suicide beyond the relatively limited research on post-traumatic stress disorder amongst first responders (Cass & Benuto, 2022; Ricciardelli et al., 2018; Stanley et al., 2016). Missing from this literature is consideration of what forms of employment are associated with suicide ideation and suicide and what questions this raises for legal responsibility, accountability, and social justice.

This paper examines what the study of work-related suicide can offer discussions concerning a “criminology of work.” It does so by documenting how work-related suicide is a seriously under-researched and undocumented phenomenon that demands a response embedded within social justice, in which the focus is on *work*-related suicide as opposed to work-related *suicide*. Informed by sociological traditions on suicide, we submit that work-related suicides in recent years reflect the changing nature of work in a globalized and digitalized economy. A social justice lens entails moving beyond a potentially narrow mental health perspective (which tends to frame suicide as an outcome of individualised psychology) to frame suicide as a collective social justice issue that reflects the rules, policies, laws and the underlying values and ideologies affecting all our working lives. It challenges us to ask questions about the nature of work in modern society, its relationship to current economic systems, and how these affect workers’ mental health. It also requires that we move beyond existing approaches to workplace mental health that tend to rely on therapeutic approaches rooted in psychological concepts of wellness, resilience and mindfulness and which focus on the individual rather than the organisation as a locus for change. These approaches often fail to address structural aspects of work and in particular, the impact of working conditions in causing the psychological distress that may lead to suicide (Song & Baicker, 2019; Davies, 2021; Fleming, 2024). There are growing calls for research on the impact of workplace organisation and relationships on suicidality and in particular, factors such as job security, working hours and work demands (Teoh, 2023). As such, in what follows we use work-related suicides as a basis for arguing that a criminology of work should focus on what to do about work (and society), not just interventions that address the impacts of work for individual workers.

This paper is organised into three sections, each of which endeavours to address the different facets of work-related suicide from a social justice perspective. We begin in the next section by outlining the limited research on work-related suicide, along with the literature that speaks to the conditions of work in globalised and digitalised economies that contribute to workers’ deteriorating mental health. The second section focuses on the lack of legal recognition of work-related suicide and how it contributes to an unjust regulatory context for workers and their families and/or loved ones. The final section explores how a social justice lens can address the multiple factors that contribute to work-related suicide and offers a possible template for the advancement of a criminology of work.

### Work-related suicide and the global economy

Recent studies call urgently for new research to examine the complex ways in which work and working conditions can exacerbate suicidality (Howard et al., 2021; Greiner & Arensman, 2022). As scholars note, work-related suicide is an under-developed area of research and there is a lack of knowledge regarding the relationship between work factors and suicide (Greiner & Arensman, 2022, WHEC 2022). Existing research is largely confined to biomedical disciplines and consists of quantitative studies that examine statistical patterns of suicide or individual risk factors. Such approaches rarely engage with the complex individual experiences and social situations that influence suicide (Mills, 2020; Chandler, 2020). Qualitative studies are limited to a small number of professions, including construction workers (Milner et al., 2017; Chan et al., 2020), emergency responders (Mars et al., 2020), doctors (Dutheil et al., 2019; Riley et al., 2021) and nurses (Peate, 2022; Riley et al., 2024). While these studies highlight risk factors in specific occupations, they cannot explain *how and why* certain occupations have high suicide risk and not others, and what the cross-sectoral work-related factors underpinning suicidality are. Our research is rooted in the following premise: to understand and prevent suicide, we need to consider the entirety of the social, economic and structural conditions affecting the individual, moving beyond a narrow and linear framing in terms of mental health alone.

In a majority of countries worldwide, work-related suicide remains a legally unrecognised, statistically undocumented and unregulated phenomenon that is excluded from the frameworks that pertain to other spheres of occupational health and safety. Work-related suicide is arguably subject to “visibility regimes” which imply that only certain social phenomena are considered relevant, important and worthy of government intervention, while others are not (Brighenti, 2010, Neumayer 2021). Such invisibilisation is not necessarily a consequence of a lack of evidence, even if research in this area is in its early stages. One recent comparative survey estimates that 10–13% of all suicides, equivalent to over 70,000 annually worldwide, are work-related. This confirms the findings of studies in the United States which identified 12% of suicides as being work-related (Peek-Asa et al., 2021), New Zealand at 12% (WorkSafe, 2024) and France at 10% (Gigonzac et al., 2021). In general, these studies identify suicides as being work-related when the death is wholly or partly caused by work or working conditions, whether structural or relational. One recent study defines this as: “a suicide in which work-related factors significantly contributed to the suicidality of the deceased” (WorkSafe, 2024). Countries including France and the USA have defined criteria for identifying suicides as potentially work-related: when a suicide occurs in the deceased person’s workplace, where the means of suicide is connected to the person’s work and/or where there is circumstantial evidence of a link to work, such as a suicide note or witness statement.

While employment can be a protective factor against suicide (Blakely et al., 2003), the highest proportion of suicides in the European Union occur among working-age adults (aged 45 to 64), accounting for 37% of all suicides. In the USA, the suicide rate within the working-age population has increased by 40% in less than two decades. While some types of work-related deaths are in decline, rates of workplace suicide are rising and increased sharply after 2007 (Tiesman et al., 2015; Peterson et al., 2020). In Japan, *karojisatsu* or suicide by overwork is treated as an urgent public health issue, and the number of people taking their own lives because of their working situation reached a peak in 2011 at 2,689 and decreased to 1,918 in 2020 (Engelmann, 2021). In the European Union, there were 39,265 (estimated) suicides amongst working-age adults in 2015 (Łyszczarz, 2021). And while we cannot assume that all suicides by working-age adults are work-related, there is a burgeoning body of literature that examines the links between work and suicide.

National statistics on suicide by occupation reveal sharply differing suicide risks across different jobs and sectors. Certain occupations for male and female employees have higher suicide rates than others, with “low-skilled” occupations and in particular, construction workers having the highest rates for men and social care and nursing having the highest rates for women (ONS Office for National Statistics, 2019; Windsor-Shellard & Gunnell, 2019). One recent report suggests that the likely correlates in occupations with high suicide risk for men are precarious employment with episodic unemployment, a predominantly male workforce, a relatively high incidence of workplace accidents, lack of consistent social support and high rates of alcohol and drug misuse (WHEC 2022). In occupations where women have a disproportionately high suicide risk, some studies point to the impact of emotional labour, depressive illnesses and burnout on mental health (Delgado et al., 2017). It is worth noting that there is a lack of data for Trans people, despite evidence that discrimination at work impacts mental health and suicide (LGTB Health and Wellbeing, 2021). Work also generates and exacerbates inequalities along social, gender and racial lines which interact with mental health and shape suicidality (Button & Marsh, 2020). For example, recent studies show how suicide rates are disproportionately high amongst migrant and undocumented workers.

Moreover, a growing body of international studies has linked suicide to psychosocial conditions of work (Waters, 2020; Howard et al., 2021; LaMontagne et al., 2024, WorkSafe, 2024). In one review of 22 independent studies of work-related suicide, Milner et al. (2018: 247) conclude: “results of this review suggest that exposure to various psychosocial job stressors was associated with elevated risk of suicide ideation, attempts and death.” Several recent studies confirm that poor working conditions and in particular precarious work, work overload, lack of control, and unregulated hours increase suicide risk (Min et al., 2015; Waters & Palmer, 2021, WHEC 2022, LaMontagne et al., 2024).

In considering the connections between work and suicide, it is essential to understand work as an integrated and multidimensional aspect of people’s lives that shapes not only material conditions, but also the very foundations of social life (Llosa et al., 2023). Since Durkheim’s *Le Suicide* (1897), we know that suicide is a socially determined phenomenon that transcends the individual and is shaped by wider structures and forces within society. Work plays a critical role in shaping an individual’s relationship to society and in particular, helps to define social belonging, collective identity and connectedness, often manifesting in the deeply problematic equating of work with people’s social worth. Indeed, the workplace is a prism that reveals the ways in which the external social world matters to individual psychological pain and suffering (Mueller et al., 2021).

The issue of work-related suicide gained widespread public attention following high-profile cases of employee suicides at several large corporations. These include Foxconn in China, one of the largest electronics manufacturers in the world and the largest employer in mainland China that makes Iphones and Apple TVs amongst other gadgets. A succession of reported suicide cases at the company culminated in 2010 where there were 18 attempted suicides and 14 deaths in a context of brutal working conditions and a harsh management culture. The cases did not lead to any legal action against the company and the employer’s response was characterised by “cosmetic efforts to fight recognition of deeper issues” including the installation of safety nets to catch falling bodies (Chan et al. 2022, 15). At Dentsu, an advertising agency in Japan, the suicide of a 24-year-old employee who was allegedly working over 70 h a week and had documented her experiences on social media, led to the prosecution of the company for a violation of labour standards. The charges targeted Dentsu as a corporate agency rather than any managers or executives at the company. It was a previous incident of suicide at the same company that had brought the issue of *karoshi* or death by overwork to public attention. At France Télécom, abusive management policies designed to cut staff costs and push employees to leave the company resulted in cases of employee suicide that culminated in 35 suicides in 2008 and 2009. These cases resulted in a landmark criminal trial that led to the prosecution of the company’s former chief executive and six other executives and managers in December 2019. Following an appeal by the defendants, France’s Supreme Court in January 2025 rejected the appeals and confirmed that the bosses where guilty of “institutional psychological bullying” for using policies that knowingly led to a “degradation of working conditions”. This is a landmark criminal case, as it is the first time that both company bosses and the company itself as a legal entity have been convicted in relation to employee suicides (Cour de cassation, 2025).

### The changing nature of work

On one level, official recognition of work-related suicide is a necessary step towards ensuring that workers receive legal protections and that there exists some form of accountability when a worker dies by suicide and where work is a contributing factor. At the same time, however, to address the conditions of work requires that we situate these developments within their broader context. Although the causal factors associated with suicide are often complex and multifactorial, the changing patterns of work in globalized and digitalized economies have become important considerations within the context of the previously discussed research on work-related suicide. Even if the exploitation of workers has been an integral part of the history of capitalism, the neoliberal turn taken by countries throughout the Global North since the 1980 s has exacerbated the problem, and in the process contributed to changing working conditions and, relatedly, workers’ deteriorating mental health. The impact of the 2007/08 global financial crisis on the global economy provides insights into the links between structural conditions, work, and mental health. Countries like Greece, hit especially hard by the crisis and subsequent austerity measures, witnessed a 40% increase in suicides from January to May 2011 compared to the previous year (Karanikolos et al., 2013). Reeve’s et al. (2014) examine “economic suicides” in Europe, the US, and Canada following the financial crisis. Acknowledging the recession did not uniformly impact mental health and suicide, the authors found a period of declining suicide rates preceding the financial crisis, followed by significant increases after its onset.

Working conditions have also changed along with shifts in the global economy – changes that have adversely impacted workers’ mental health. The near decimation of private sector unions, the war on regulators and regulatory agencies, privatization, “free” trade deals, downsizing and outsourcing have all increased employees’ vulnerability *vis a vis* employers (Sayer, 2016; Tombs, 2016). Changes in welfare, employment insurance and numerous other entitlements under the previous Keynesian welfare state has added yet more pressure by removing alternate sources of income when they were/are most needed. With globalization taking away jobs, automation replacing many that remain, and neoliberalism removing most sources of state support, the working and employee classes are increasingly desperate, forced to accept whatever work and wage conditions employers are offering (Stone, 2006). It is noteworthy that some studies suggest that work-related suicide constitutes a new phenomenon in historical terms, reflecting the specific features of capitalism at the present historical juncture (Clot & Gollac, 2017; Waters, 2020). Leading international specialist Christophe Dejours noted that in the French context, documented cases of work-related suicide prior to the 1990s were relatively rare and generally confined to the agricultural sector and linked to factors of social isolation (Dejours & Bègue, 2009).

Neoliberalism, with its commitment to private enterprise, maximal corporate profits, minimal state regulation of business, and hyper-individualism that prioritizes personal wealth and accomplishment over collective solidarity, has profoundly changed employment relations (Ilcan, 2009; Peck, 2010; Peck & Theodore, 2012; Sennett, 1998). In addition to stagnating wages, leaving workers in debt as they seek other means to augment their dwindling income, work is increasingly demanding, precarious, part-time, and low paying (Bittle & Snider, 2018; Cooper & Whyte, 2017; Harvey, 2014; Stone, 2006). Precarious work alone contributes to increased “…stress, anxiety, sleep disorders, burnout and in some cases, suicide” (Waters et al., 2016: 231). Coupled with this is an erosion of workers’ rights and protections, including reduced union representation, which puts workers in difficult positions when attempting to realize their already limited protections (Waters et al., 2016: 231). Increased pressures at work that can result in suicide are thus part of the growing realities that many people face under austerity-driven, neoliberal capitalism (Cooper & Whyte, 2017).

Cementing these practices is a neoliberal ethos which promotes “flexibility” and economic “restraint” (read: austerity) for employees, urging workers to accept a future without full-time, permanent employment and believe that changing jobs to suit employers is a “choice”, part of our collective responsibility to “grow” the capitalist economy (Sayer, 2016; Sennett, 1998). This dramatic shift in labour markets has a distinctively moral character; it fosters a “cultural political economy” where private enterprise is seen as an inherent good (Tombs, 2016: 36–37). This “marketization of all social relations” applies a raw economic rationality to all human activity (Whyte & Wiegratz, 2016: 10). Today there is no such thing as job shortages, just “inadequate” individuals who, despite occupying very different and increasingly unequal social locations, make bad decisions (Sayer, 2016).

Jaffe (2021) notes that this ethos is indicative of the changing nature of work under neoliberal capitalism, particularly the deteriorating conditions of work (i.e., precarity, poor pay, long hours, increasing expectations) and the illusion of choice that accompanies contemporary work arrangements. Failure to work or the failure to love your work is seen as an individual failing – you just have not done enough to succeed at your job, or you have failed to get the necessary training or education to get the job you want. This mantra of ‘love your work’ cuts across all forms of employment, drawing from a narrative that blurs the lines between work and personal lives, positioning work as a family-like arrangement. Of course, lurking in the background is the profoundly transformative program of privatization that has been integral to the neoliberal shift. Privatization not only makes workers expendable (easily hired, fired and controlled), which serves the interests of employers, but it has generated increased rates of depression and anxiety among workers. What’s more, all the rhetoric about loving your work obscures the fundamental coercion that is at the “heart of the labor relation” (Jaffe, 2021: 9). As Jaffe argues, “Neoliberalism relies on the labour of love ideology to cover up the coercion that was in fact required to push people into the workplace at the origin of capitalism” (Jaffe, 2021: 10; also see Glasbeek, 2024).

Jaffe notes that work plays a fundamental role in shaping who we are, or “how to be.” However, as she also points out, “…changes in the shape of the workplace, in the shape of capitalism itself, have changed our expectations for what our lives will be like, for where and how we will find fulfillment. The concept of a “good” job is one that has changed over time and through struggle, a point we would do well to remember” (Jaffe, 2021: 10). As such, instead of arguing that work insulates people from poor mental health, we need to question the very basis of work – its socially constructed arrangement in modern capitalist society. “The idea that work should be a source of fulfilment has become common sense in our world, to the extent that saying otherwise is an act of rebellion” (Jaffe, 2021: 11).

Within this context pursuing individual life goals with little regard for social commitments, collective responsibilities or societal impact is simply what one must do to get ahead. Working multiple jobs, changing careers, countries and communities at a moment’s notice, working more for less are all part of ‘doing what it takes’ to realize the opportunities that are (we are told) there for the neoliberal citizen. In this framing, failure to capitalize on what the economy offers is an individual flaw, not a constraint imposed by shifts in globalized trade patterns or the political economy. Workers’ deteriorating mental health, including cases of work-related suicide, is thus a symptom of neoliberalism’s methodical reengineering of labour relations in ways that favour private enterprise and translate market values into societal values (Tombs & Whyte, 2015: 30; Whyte & Wiegratz, 2016).

The relevance of the preceding for discussions of work-related suicide is that these are the very conditions in which workers’ mental health has suffered and which can and does lead to suicide ideation and suicide. Once again, while the factors associated with an individual’s decision to complete suicide are often complex, the still-evolving research on work-related suicide suggests it is vital to consider the changing nature of work and its impact on workers’ mental health. Working conditions have deteriorated massively in recent decades, along with stagnated wages and increasing costs of living (which can also be seen as working conditions given the prominent role that corporations have played in lobbying for a securing these conditions), and workers have paid the price. “The simple reality of work under capitalism is that the worker doesn’t control much of anything on the job. The fact doesn’t change if the job is pleasant, or if wages increase by a dollar an hour or by ten dollars and hour. The concept of alienation isn’t about your feelings; it’s about whether you have the power where and how hard you will work, and whether you will control the thing you make or the service you provide” (Jaffe, 2021: 10). It is within this context that we find limited research, legal recognition, and compensation for work-related suicide.

### Legal and statistical recognition

Despite emerging research and statistical evidence on work-related suicide, there have been few developments in national or international policy to tackle this phenomenon. Data can serve as an instrument of social justice and a means to transform a previously hidden or unnamed social issue into a visible and actionable problem by bringing it into the “sightlines of justice”. By constituting an issue as a recognisable social problem, data can also make others accountable: “Numbers and justice have long kept company, as the paired words counting and accounting attest. If you can count something, you can also account for it” (Jasanoff, 2017, p. 1). Yet few countries collect data on work-related suicide. In the US, the Labour Statistics Bureau has collected data on work-related suicide since 1992, although some have criticised the data for inaccuracy and under-reporting. In Japan, data is collected by the police, who can record work or working conditions as a factor in registering the death. In France, data exists from a variety of sources and the government has put in place an initiative to centralise and improve data collection. However, in most countries, data is limited to suicide rates by occupation (rather than where work is a causal factor in the suicide). In Australia, government ministers have recently agreed to amend Workplace Health and Safety laws to include suicide and attempted suicide as reportable work-related incidents. Under the terms of these amendments, employers will be required immediately to notify regulators of any work-related or suspected work-related suicide or attempted suicide that has potential links to work or the work environment (DWF 2024).

However, in a vast majority of countries worldwide, work-related suicide has no statistical or legal recognition and is excluded from the legal protections that pertain to other areas of work-related injury or death. The UK context is instructive in this regard in that work-related suicide has very limited recognition and it is extremely rare for cases to reach the courts. While extant UK legislation relevant to workplace health and safety are sufficiently broad in scope to include work-related suicide, in practice they have yet to be used. For instance, the *Health and Safety at Work Act 1974* imposes on employers a general duty of care to “ensure, so far as is reasonably practicable, the health, safety and welfare of all… employees”, and a failure to do so is considered a criminal offence. The notion of health and safety as set out in this law, was devised in relation to injuries or death arising from physical accidents, but it has also been widely interpreted as covering mental health within its scope. In addition, the 2010 *Equality Act* makes it unlawful for an employer to discriminate against an employee on the grounds of a mental illness. Hence, an employer can be held liable for discrimination where they do not make sufficient adjustments to work to take account of an employee’s disability. Under the terms of this legislation, mental ill-health is framed as a pre-existing condition that a person brings into the workplace, rather than an issue that potentially arises from the impact of workplace structures, conditions or practices on the employee (Waters, 2022). While this law was recently used in a landmark case to take legal action against a university for discrimination in the case of a student suicide, it has never been used in a case of work-related suicide (D’Alton-Harrison, 2024).^1^

Alongside this legislative framework, responsibility for protecting workplace mental health lies with the UK regulator, the Health and Safety Executive which is responsible for preventing “workplace death, injury or ill health” and this includes “taking enforcement action to prevent harm and hold those who break the law to account”. While employers are legally obliged to report all work-related ‘accidents’ and deaths to the HSE, its guidelines specifically exclude suicide: “All deaths to workers and non-workers, *with the exception of suicides*, must be reported if they arise from a work-related accident” (HSE). Since work-related suicides are not reported, they are not investigated by the HSE and there are no legal duties on the employer to make any changes in the workplace following an incident of work-related suicide (Waters, 2022).

In the UK, cases of work-related suicide are therefore tackled outside of a health and safety framework within the coronial system where they may be subject to a public inquest. Coroners have a duty to take action to prevent deaths and may decide to issue a Prevention of Future Deaths (PFD) Report to raise concerns that may lead to preventative measures. In practice, use of PFD reports in the case of work-related death is very rare. According to the Preventable Deaths Tracker, between 2013 and 2024, only 55 PFD reports were issued to the Health and Safety Executive covering all work-related fatalities (compared with 727 issued to the Department of Health and Social Care during the same period) (Preventable Deaths Tracker). Even where PFD reports are issued, they are not legally enforceable, and a coroner has no powers to monitor whether the employer acts on them on not. Some bereaved family groups have criticised the coroners’ system as secretive, opaque and unaccountable, relying on considerable discretionary powers of the individual coroner (CSPAAG, 2006).

While the UK scenario is indicative of the lack of recognition and legal protections associated with work-related suicide, there are nevertheless some exceptions. In Japan, for instance, the 2014 Act on Promotion of Preventive Measures against *Karôshi* makes the government responsible for promoting measures to prevent injury or death at work, including: “death by suicide due to a mental disorder that is brought on by an intense psychological burden at work.”^2^ In a recent case the suicide of a man who held two jobs was recognized as a work-related death in 2024 based on the combined psychological stress he suffered from both workplaces (The Asahi Shimbun 2024). Work-related suicide also has limited legal recognition in some European countries including Belgium, Spain and Italy. Countries such as Finland strictly rule out recognition of suicide as a potential accident at work (Eurogip, 2023). Developments in case-law across different countries have allowed employers to be held accountable in cases of work-related suicide. In Spain, a court recently recognised a suicide that took place outside of the workplace as work-related because of circumstances linking the suicide to work (L&E Global 2023). Employer liability in cases of employee suicide has been addressed in recent court cases in India (Mondaq, 2025).

The French context is also distinctive in that work-related suicide is recognised under social security law as a potential “occupational accident” incurring the legal and financial responsibility of the employer. A suicide in France is presumed to be work-related and subject to further investigation when it occurs in the workplace, on the journey to and from work, where there is a material link to work (suicide letter or witness statement), where a work implement or tool was used (such as a firearm or medication), or when work clothes are worn at the time of the suicide. Any suicide that occurs in the workplace in France is automatically investigated as an occupational accident and the burden of proof is on the employer to prove that the suicide is not work-related. Even in cases where a suicide takes place outside of work, it is still investigated as work-related where the employee (in an attempted suicide) or the family can prove a causal link to work. According to one study, approximately half of the suicide cases brought to the social security authorities by family members are officially recognised as work-related (ONS, 2014, 69).

Within the terms of French labour law, an employer can also be held legally responsible for not fulfilling their health and safety obligations in cases of work-related suicide. Unlike many other jurisdictions, French law makes specific reference to mental health and the employer must take ‘the measures necessary to ensure the safety and protect the *physical and mental health* of the workers’ (Article L.4121-1 of Labour Code). These measures include action to prevent occupational risks, information and training action ensuring a workplace organization that adapts to changing circumstances and conditions. The approach is based on the application of general prevention of harm principles set out in Article L. 4121-2, such as adapting work to the individual, tackling risks at source, preventing the risks of workplace bullying and harassment. While there is no pre-existing definition of work-related suicide within French labour law, courts have the power to determine the causal link between work and suicide based on the evidence presented.

Recognition of suicide within French legislation has led to a number of high-profile cases, notably the criminal prosecution of company executives at France Télécom. Alongside this landmark case, other large French companies have been held responsible for gross negligence under the terms of social security law whereby, the employer was or should have been aware of the danger to which the employee was exposed and did not take the necessary measures to protect them. These include Renault, La Poste and SNCF. According to one account, 97 employees at La Poste took their own lives or attempted suicide between 2009 and 2013 (Burgi & Postier, 2013). Employers have also been held responsible for gross negligence in cases of attempted suicide (Robert, 2023).

Regardless of these exceptions, the lack of recognition, regulation and prevention of work-related suicide is a key source of injustice for workers and their families in most countries globally. On one hand, workers across all jobs and sectors are exposed to enduring and preventable suicide risks. However, in most instances, there is no legal requirements for employers to implement preventative measures following a suicide, meaning the causal factors that may have led to one suicide may continue to pose an ongoing risk to other workers in the same company or workplace. Scholars suggest that work-related suicide is not an isolated event but the tip of an iceberg, signalling a broader mental health crisis across the whole workplace: “A single suicide in a company constitutes a de facto problem affecting the entire workplace community to the event that it reveals a profound deterioration in the human and social fabric of work” (Dejours & Bègue, 2009, p. 14). One recent UK study examined a cluster of four suicides occurring in the same workplace and in the same year. None of the suicides was officially reported and subject to an investigation by the health and safety regulator. No duties were placed on the employer to act following any of the suicides (Waters & Palmer, 2021). Yet families who lose the salary of a loved one may be left without financial compensation. Furthermore, when challenging companies through litigation, they are rarely entitled to legal representation or redress.

Recognising the complexity and multiplicity of factors that may contribute to suicide can occur simultaneously alongside invoking government and corporate culpability (Mills, 2018) – evident in organising and campaigning led by bereaved families (see for example, Families Against Corporate Killing, FACK). Mark Button argues for a political-institutional approach that considers how the formation of vulnerability to suicide is shaped by politics and by the impact of differing ‘suicidal regimes’ (Button, 2020 87). An international social justice approach can therefore help to elucidate ways in which policy frameworks on work-related suicide are shaped by specific and contingent political decisions, rather than self-evident psychological truths regarding the nature of suicide and its motivations. Furthermore, such a perspective can provide valuable alternative models for responding to suicide that could be effectively used elsewhere. The final section of this paper provides further insight into this social justice lens and how it relates to both work-related suicide and, potentially, a criminology of work.

### An international social justice perspective

Dominant medical and ‘psy’ (psychiatric, psychological, and psychotherapeutic) approaches to suicide tend to frame it as an issue of individual ‘mental illness’ (Marsh, 2010). Interventions are often individual or at best interpersonal and often focussed on improving mental health. This treats suicide as apolitical – with no implications for structural or systemic accountability and change; and because alternative interpretations of suicide (e.g. as contextualised, as historically and culturally contingent, or as a method of resistance) are marginalised or silenced (Marsh, 2010: 43 and 219). Prevalent biomedical approaches have shaped the ways in which suicide is identified, recognised and understood and in turn, have influenced the frameworks by which solutions to suicide are conceptualised and organised (Marzetti et al., 2023).

Governments, for instance, tend to approach suicide as a complex issue that resides in the mind and in the personal and emotional lives of individuals (Button, 2020), evident in that on the rare occasions where people’s suicides are investigated, they are often treated as individual ‘cases’, obscuring wider patterns (Mills, 2018). As Mills (2018) points out in relation to suicides linked to the UK welfare system (relevant here because of the corelation between suicide and being found, as part of a disability benefits assessment, to be ‘fit for work’), while people’s decision to die by suicide may well be complex, the mobilisation of this complexity delimits recognition of government and corporate culpability’. Suicide is so strongly equated in public discourse with individual mental health that naming certain deaths as suicide obscures the ways structures and systems, including workplaces, make lives unliveable, enabling denials of responsibility through blaming the victims.

Differently, Critical Suicide Studies widens our understanding of suicide – not only to counter the limitations of narrow reductionist dominant approaches but to document and explore alternatives ways of understanding that can inform collective action (Chandler et al., 2022; Jaworski & Marsh, 2024; White et al., 2016). A key strand of scholarship and praxis within Critical Suicide Studies frames suicide as a social justice issue – emphasising the contexts (political, economic, social, cultural and historical) and social forces that shape suicide, and in so doing, creating space for collective and political accountability and action (Marsh 2014, 2022; White et al., 2016). Within this literature, suicide is understood as an issue of collective social justice tied to ‘the distribution of primary goods within a political system’ (Button, 2016:274) and shaped by ‘structural, discursive, material and social-historical relations that both produce and reproduce colonial violence, entrench inequalities, and create vulnerabilities amongst some groups and not others’ (White, 2017: 472–473).

Within the growing literature, social justice approaches to suicide tend to do a number of things:

1. They seek to reject narrow reductionist apolitical ‘psy’ explanations of suicide as ‘mental illness’ and recognise that these explanations may be a form of epistemic injustice (i.e. in the way they question the reality of people’s experiences and their status as knowers) (Fricker, 2007; Marsh, 2010; Mills, 2018; White et al., 2016).
2. They take seriously injustice as a cause or contribution to suicide – i.e. they ‘structure into our analysis of a person’s death the context of social injustice in which they lived’ (Reynolds, 2016:170).
3. They draw upon and move further than a social determinants approach to suicide by attending to how socio-political forces (such as, racism, ableism, colonialism etc.) shape social inequalities, directly and systematically impacting rates of suicide (Marsh, 2020).
4. They orient towards collective action and social change (White, 2017) in order to create more just social arrangements.
5. They use methodologies for knowledge production that are sensitive to complexity, creative, and rooted in lived experience (Marsh, 2020), where experiential knowledge is key in countering the decontextualization evident in much mainstream suicidology (White et al., 2016; Webb, 2010).

Understanding suicide as an issue for social justice is more than conceptual and is evident in the political activism and campaigning of the families and friends of many of those who have died through suicide, including by suicide related to work (see family campaigning in relation to UK welfare reform and suicide, Mills, 2018). Thus, social justice approaches are about understanding social causality (i.e. how injustice creates the conditions for suicide) and about driving action for accountability and real change that prevents such deaths in future, to move towards a more ‘hopeful, life-affirming, and just future’ (White, 2017: 472). While these are not easy issues to confront, our point in drawing upon a social justice lens is to situate these efforts within their broader context – to ensure they are buttressed by even broader struggles around the nature of work in capitalist societies, or, indeed, the profound harms and inequalities of capitalism in general. The workplace has long been recognised as a site for structural inequalities based along social, gender and ethnic lines. Our aim is to integrate an understanding of how structural inequalities interact with conditions of work to create a differential vulnerability to suicide amongst individuals and groups.

When it comes to contemplating the relevance of a social justice framework for the criminology of work, it is important to note the synergies of this approach with extant criminological literature that employs a social harm perspective. Rooted in critical criminology, social harm scholars endeavour to move beyond the narrow concept of ‘crime’, which leaves unchallenged a “significant amount of suffering and injury (Leighton & Wyatt, 2021: 2), to consider the range of physical, financial, and emotional harms that people experience throughout their lives (Dorling et al., 2008; Canning & Tombs 2012). As Canning and Tombs (2012: 1) note, it is an approach that builds from the discipline of zemiology, which “…seeks to unearth harmful structures, policies, decisions and practices, evidences the impacts that they have and thus generates sustainable and radical changes so that they may be mitigated or eradicated.” For instance, Dorling et al., criticize mainstream criminology for perpetuating the myth of crime – routinely ignoring the fact that crime has no ontological reality – on route to advocating for a perspective that scrutinizes a more complete range of “detrimental activities” and various forms of danger that people routinely experience in contemporary society. Social harm scholars also invite us to consider state responsibility for matters that include the failure to address social and economic inequalities, or corporate responsibility for polluting the environment, sickening and killing workers and the public, or destroying the economy (Dorling et al., 2008; Hillyard & Tombs, 2004, 2007). According to Dorling et al., considering a broader range of harms than what is commonly addressed through the lens of crime challenges us to think critically about the conditions that give rise harmful acts in society, or what they refer to as the “social origins of harm”. In the process, it brings to the fore the experiences of those who are victimized by social harms than might otherwise be the case if we adhered to strict legal definitions of crime. From our perspective, a social justice/social harm lens is thus essential for any analysis of work-related suicide that attempts to extend beyond dominant biomedical models of understanding the phenomenon and is also instructive for any discussions of what might constitute the focus of a criminology of work.

Grounding work-related suicide in social justice does more than increase awareness of how social context and structural conditions shape both suicidality and working conditions. It pays attention to the forces shaping these conditions, and to who benefits and profits from them – the governments and corporations invested in the current status quo. A social justice approach to work-related suicide also foregrounds principles of accountability, prevention and justice for suicidal individuals, their families and workers across all sectors and occupations. It frames suicide as a matter of collective social justice and calls for action to ensure safe workplaces, to protect the right to life and prevent suicide deaths.

When applied to work-related suicide, a social justice approach might be based on three core principles. First, it argues for work-related suicide to be accorded **legal recognition** as a terrain for the protection and assertion of individual rights and in particular, the right to life and safe working conditions. It recognises the importance of legal and policy instruments to ensure safe working conditions, create duties on employers to prevent suicide and to enhance accountability and justice. Furthermore, adopting an international social justice approach can help to elucidate the ways in which policy frameworks on work-related suicide are shaped by specific and contingent political decisions made by particular governments (often with significant influence from the business sector), rather than self-evident psychological truths regarding the nature of suicide and its motivations.

Second, a social justice perspective attends to the **social and structural conditions** of work and the ways in which they may create a differential vulnerability to suicide amongst individuals and groups. It recognises the ways in which work can generate and exacerbate inequalities along social, gender and ethnic lines which interact with mental health and shape suicidality (Button & Marsh, 2020). Such an approach bridges connections between suicide research and labour studies and, in particular, emerging scholarship on the relationship between working conditions and suicide.

Third, a social justice approach calls for **access to justice** for bereaved families, so that they are accorded instruments of legal and financial redress in the aftermath of the suicide of a loved one. Studies show that families often experience a profound sense of injustice in the aftermath of a work-related suicide whereby the causes of death are not always fully investigated, they are denied the legal means to challenge organisations and may be left without financial compensation (Waters & Palmer, 2021).^3^

Social justice can also be conceived in addition to or outside of and beyond the more legalistic parameters outlined above. Many families bereaved due to state violence, experience little to no justice within a criminal legal system that largely serves the interests of those with power. If there is to be justice and healing, then for many this does not come solely or at all through proximity to the State. For example, the Deaths by Welfare project, grounded in a social justice approach to understanding suicides linked to the policies of the UK welfare state (and co-designed with people who experience suicidality in relation to the these policies), shows that people hold very different understandings of justice – both within, against and outside of the State.^4^ While some families campaign for government accountability and change, many experience justice, and healing, through solidarity, community, care and witnessing. However we are deeply conditioned to equate justice with punishment (i.e., that justice is about criminal penalties and imprisonment), and it takes significant resourcing to shift our thinking, including many bereaved families and people with lived experience, to build and strengthen community networks that provide solidarity to individuals and families and move toward more expansive practises of justice – those that are reparative, restorative, and/or transformative.

Much can be learned here from other domains of advocacy, which have identified the need for structural change, while at the same time recognising the limitations of purely legal approaches to justice. A key example is activism and scholarship in the space of prison abolition. Faced with a sense that “nothing can be done until the state is overthrown,” some abolitionists have argued for *non-reformist reforms*—changes that confront material conditions in the short term, while also being consistent with, and building towards, larger collective transformational demands (Kaba, cited in Duda, 2017; and see the work of Critical Resistance). Non-reformist reforms would need to address both government regulation and private companies, as part of larger collective demands to challenge the very nature of work in contemporary society and the myriad ways it contributes to workers’ deteriorating mental health. Doing so entails allied groups working together (for instance, FACK, unions, groups advocating for precarious and marginalised workers) to help workers gain better control over their working conditions. The ultimate aim of such forms of social advocacy is to challenge decades of (neoliberal) transformation of workplaces that have favoured employers at the expense of workers’ health and safety. They acknowledge both the limits of the state in regulating employers (particularly the powerful) and the importance of resistance and collective wellbeing. After all, the state remains a capitalist state, which means that struggles for official recognition of work-related suicide, along with related demands for new laws to hold employers to account, must also acknowledge that states tend to regulate in ways that favours the interests of business (Tombs & Whyte, 2015). It means the underpinning conditions of work in contemporary capitalist society that contribute to workers’ deteriorating mental health are likely to remain intact regardless of any legislative innovations.

### Conclusion: what to do about work?

This paper examined work-related suicide as an under-researched and unrecognized phenomenon that requires a social justice lens. Work-related suicide has emerged as a growing issue of concern in recent years within the context of neoliberalised and globalised economies that prioritize maximal corporate profits and worker ‘efficiencies’ at the expense of workers’ mental health. As work has become more precarious and demanding, as workers are required to do more with less, and as the failure to ‘love your job’ is seen as a personal deficiency (Jaffe, 2021), some scholars and policy makers have started to acknowledge that work and working conditions play a significant role in why some workers either contemplate, attempt, or complete suicide. There are few legal and policy mechanisms in response to the phenomenon, creating an unjust scenario in which those affected by work-related suicide, including bereaved families, receive no official recognition of the harms they experienced, and therefore no steps are taken to determine if employers have any legal or compensatory liabilities. In the workplace, the conditions that may have led to one suicide can continue to pose a serious risk to all other employees, as there is no legal obligation on employers to act or put preventative measures in place.

Employing a social justice lens, we argued that, as a phenomenon, work-related suicide raises fundamental questions about the nature of work in modern society, the values underlying current economic systems, and, indeed, about the very societies in which we live. From our perspective, a social justice lens entails moving beyond a solely biomedical or mental health perspective to frame suicide as a collective social justice issue that reflects the rules, policies and laws that affect all our working lives. It is about exploring ways to understand and address *work*-related suicide, as opposed to work-related *suicide*. We have suggested that using a social justice lens in relation to work-related suicide would involve concrete actions to recognise work-related suicide in legal and policy terms, to incorporate social and structural conditions into our analysis of suicide, and to increase access to justice for bereaved families.

Research and policy development on work-related suicide remains at its relative infancy. It is our hope that this paper makes a modest contribution to ongoing discussions about this important issue, particularly as it relates to advancing a social justice perspective. It is also our hope that our work contributes to burgeoning discussions about the prospects of a criminology of work by expanding the parameters of what is considered as recognisable and actionable within the terms of the law and as it relates to broader social change.

## Data Availability

Not applicable.

## Declarations

1 There has been one UK case of civil liability for a work-related suicide. In Corr vs. IBC Vehicles, the widow of a maintenance engineer was successful in her case against Vauxhall Motors following the suicide of her husband in 2002 who suffered depression following a serious physical injury incurred several years previously while working at the company. While the employer contested their duty of care, arguing that it did not extend to protecting an employee against suicide, the Court of Appeal found that the victim’s depression was a foreseeable consequence of the accident and that the suicide itself was therefore foreseeable (Freckleton 2009). While this is a landmark case that has established potential employer civil liability with respect to an employee suicide, it has not been followed by any subsequent prosecution cases for work-related suicide.

2 See Article 2 of *An Act p*romoting Measures to Prevent Death and Injury from Overwork. https://www.japaneselawtranslation.go.jp/en/laws/view/3258/en.

3 Work-related suicide is only eligible for workers’ compensation in a small number of jurisdictions around the world, including include France, Japan, Canada, and Australia (LaMontagne et al., 2024).

4 See: https://deathsbywelfare.org/.

## References

[R1] Almond P (2010). Corporate manslaughter and regulator reform.

[R2] Bittle S (2012). Still dying for a living: Corporate criminal liability after the Westray mine disaster.

[R3] Bittle S, Snider L (2018). How employers steal from employees: The untold story. Social Justice: A Journal of Crime Conflict and World order.

[R4] Blakely TA, Collings SCD, Atkinson J (2003). Unemployment and suicide. Evidence for a causal association?. Journal of Epidemiology and Community Health.

[R5] Brighenti AB (2010). Visibility in Social Theory and Social Research.

[R6] Bruckert C, Hannem S (2013). Rethinking the prostitution debates: Transcending structural stigma in systemic responses to sex work. Canadian Journal of Law and Society.

[R7] Burgi N, Postier A (2013). Le Monde diplomatique.

[R8] Button ME (2016). Suicide and social justice: Toward a political approach to suicide. Political Research Quarterly.

[R9] Button ME, Marsh I (2020). Suicide and social Justice New perspectives on the politics of suicide and suicide prevention.

[R10] Canning V, Tombs S (2012). From social harm to Zemiology: A critical introduction.

[R11] Cass JB, Benuto LT (2022). Work-related traumatic stress spillover in first responder families: A systemic review of the literature. Psychological Trauma: Theory, Research, Practice, and Policy.

[R12] Chan APC, Nwaogu JM, Naslund JA (2020). Mental ill-health risk factors in the construction industry: Systematic review. Journal of Construction Engineering and Management.

[R13] Chan J, Distelhorst G, Kessler D, Lee J, Martin-Ortega O, Pawlicki P, Selden M, Selwyn B (2022). After the foxconn suicides in China: A roundtable on labor, the state and civil society in global electronics. Critical Sociology.

[R14] Chandler A, Button ME, Marsh I (2020). Suicide and social justice New perspectives on the politics of suicide and suicide prevention.

[R15] Chandler A, Cover R, Fitzpatrick SJ (2022). Critical suicide studies, between methodology and ethics: Introduction. Health (San Francisco).

[R16] Clot Y, Gollac M (2017). Le travail peut-il devenir supportable?.

[R17] Cooper V, Whyte D (2017). The violence of austerity.

[R18] Cour de cassation (2025). Chambre criminelle - pourvoi n° 22-87.145.

[R19] CSPAAG (2006). Evidence submitted by the Coroners Services Public Accountability Action Group (CSPAAG) to UK Select Committee on Constitutional Affairs.

[R20] D’Alton-Harrison R (2024). Protecting belonging through anticipation: Duty, duty, duty?. Law Teacher.

[R21] Davies J (2021). Sedated: How modern capitalism created our mental health crisis.

[R22] Dejours C, Bègue F (2009). Suicide et travail: Que faire?.

[R23] Delgado C, Upton D, Ranse K, Furness T, Foster K (2017). Nurses’ resilience and the emotional labour of nursing work: An integrative review of empirical literature. International Journal of Nursing Studies.

[R24] Dorling D, Gordon D, Hillyard P, Pantazis C, Pemberton S, Tombs S (2008). Criminal obsessions: Why harm matters more than crime.

[R25] Dutheil F (2019). Suicide among physicians and health-care workers: A systematic review and meta-analysis. PLoS One.

[R26] Engelmann J (2021). Number of suicides related to problems at work in Japan from 2011 to 2020, Statista.

[R27] Eurogip (2023). Recognition and compensation of work-related mental disorders in Europe.

[R28] Fleming WJ (2024). Employee well-being outcomes from individual-level mental health interventions: Cross-sectional evidence from the United Kingdom. Industrial Relations Journal.

[R29] Fricker M (2007). Epistemic injustice: Power and the ethics of knowing.

[R30] Gigonzac V, Khireddine-Medouni I, Chan-Chee C, Chérié-Challine L (2021). Surveillance des suicides En Lien potential avec Le travail.

[R31] Glasbeek H (2024). Law at work: The coercion and Co-optation of the working class.

[R32] Greiner BA, Arensman E (2022). The role of work in suicidal behavior - uncovering priorities for research and prevention. Scandinavian Journal of Work Environment & Health.

[R33] Harvey D (2014). Seventeen contradictions and the end of capitalism.

[R34] Hillyard P, Tombs S, Hillyard P, Pantazis C, Tombs S, Gordon D (2004). Beyond criminology: Taking harm seriously.

[R35] Howard MC, Follmer KB, Smith MB, Tucker RP, Van Zandt EC (2021). Work and suicide: An interdisciplinary systematic literature review. Journal of Organizational Behavior.

[R36] Ilcan S (2009). Privatizing responsibility: Public sector reform under neoliberal government. Canadian Review of Sociology.

[R37] Jaffe S (2021). Work won’t love you back: How devotion to our jobs keeps Us exploited, exhauster, and alone.

[R38] Jasanoff S (2017). Virtual, visible, and actionable: Data assemblages and the sightlines of justice. Big Data & Society.

[R39] Jaworski K, Marsh I (2024). Reframing suicide: The development of critical suicide studies.

[R40] Kapuscinski CA, Braithwaite J, Chapman B (1998). Unemployment and crime: Toward resolving the paradox. Journal of Quantitative Criminology.

[R41] Karanikolos M, Mladovsky P, Cylus J, Thomson S, Basu S, Mackenbach D, McKee M (2013). Financial crisis, austerity, and health in Europe. Lancet.

[R42] LaMontagne AD, Aberg M, Blomquist S, Glozier N, Grenier BA, Gullestrup J, Harvey SB, Kyron MJ, Ida EH, Madsen L, Magnusson Hanson H (2024). Work-related suicide: Evolving understandings of etiology & Intervention. Am J Ind Med.

[R43] Leighton P, Wyatt T, Davies P, Leighton P, Wyatt T (2021). The palgrave handbook of social harm Palgrave studies in victims and victimology.

[R44] LGTB Health and Wellbeing (2021). Trans People and Work: Survey Report.

[R45] Llosa JA, Agulló-Tomás E, Menéndez-Espina S, Oliveros B (2023). Revisiting the work-suicide link: Renewed evidence and models of analysis in workplace contexts. Frontiers in Psychology.

[R46] Łyszczarz B (2021). Production losses attributable to suicide deaths in European union. BMC Public Health.

[R47] Mars B, Hird K, Bell F, James C, Gunnell D (2020). Suicide among ambulance service staff: A review of coroner and employment records. British Paramedic Journal.

[R48] Marsh I (2010). Suicide: Foucault, History, truth.

[R49] Marsh I (2020). The social production of psychocentric knowledge in suicidology. Social Epistemology.

[R50] Marzetti H, Chandler A, Jordan A, Oaten A (2023). The politics of LGBT+ suicide and suicide prevention in the UK: Risk, responsibility and rhetoric. Culture, Health & Sexuality.

[R51] Mills C (2018). Dead people don’t claim’: A psychopolitical autopsy of UK austerity suicide. Critical Social Policy.

[R52] Mills C, Button ME, Marsh I (2020). Suicide and Social Justice New Perspectives on the Politics of Suicide and Suicide Prevention.

[R53] Milner A, Maheen H, Currier D, LaMontagne AD (2017). Male suicide among construction workers in Australia: A qualitative analysis of the major stressors precipitating death. BMC Public Health.

[R54] Milner A, Witt K, LaMontagne AD, Niedhammer I (2018). Psychosocial job stressors and suicidality: A meta-analysis and systematic review. Occupational and Environmental Medicine.

[R55] Min K (2015). Precarious employment and the risk of suicidal ideation and suicide attempts. Preventive Medicine.

[R56] Mondaq (2025). Employer liability in employee harassment and suicide cases: Legal insights and preventive measures.

[R57] Mueller AS, Abrutyn S, Pescosolido B, Diefendorf S (2021). The social roots of suicide: Theorizing how the external social world matters to suicide and suicide prevention. Frontiers in Psychology.

[R58] Neumayer C, Rossi L, Struthers DM (2021). Invisible data: A framework for understanding visibility processes in social media data. Social Media + Society.

[R59] ONS Observatoire National du Suicide (2014). Suicide. Etats des lieux des connaissances et perspectives de recherche (novembre 2014). Suicide Etat des lieux des connaissances et perspectives de recherche - Ministère du Travail, de la Santé, des Solidarités et des Familles.

[R60] ONS Office for National Statistics (2019). 2019 Suicide by occupation, England and Wales, 2011-2019 registrations.

[R61] Peate I (2022). Suicide among female nurses. British Journal of Nursing.

[R62] Peck J (2010). Constructions of neoliberal reason.

[R63] Peck J, Theodore N (2012). Politicizing contingent work: Countering neoliberal labor market regulation. From the Bottom Up?. The South Atlantic Quarterly.

[R64] Peek-Asa C, Zhang L, Hamann C, Davis J, Casteel C (2021). The prevalence of work-related suicides varies by reporting source from the National Violent Death Reporting System. American Journal of Industrial Medicine.

[R65] Peterson C, Sussell A, Li J, Schumacher PK, Yeoman K, Stone DM (2020). Suicide rates by industry and occupation – national violent death reporting system, 32 states, 2016, MMWR. Morbidity and Mortality Weekly Report.

[R66] Raphael S, Winter-Ebmer R (2001). Identifying the effect of unemployment on crime. The Journal of Law & Economics.

[R67] Reeves A, McKee M, Stuckler D (2014). Economic suicides in the great recession in Europe and North America. The British Journal of Psychiatry.

[R68] Reynolds V, White J, Marsh I, Kral MJ, Morris J (2016). Critical suicidology: Transforming suicide research and prevention for the 21st century.

[R69] Ricciardelli R, Carleton RN, Groll D, Cramm H (2018). Qualitatively unpacking Canadian public safety personnel experiences of trauma and their well-being. Canadian Journal of Criminology and Criminal Justice.

[R70] Riley R, Buszewicz M, Kokab F, Gopfert A, Taylor AK, Van Hove M, Martin J, Appleby L, ChewGraham C (2021). Sources of work-related psychological distress experienced by UK-wide foundation and junior doctors: A qualitative study. British Medical Journal Open.

[R71] Riley R, Causer H, Patrick L (2024). Why are dominant suicidology approaches failing nurses? A call for a feminist critical suicidology perspective. Journal of Advanced Nursing.

[R72] Robert A (2023). Suicide et faute inexcusable de l’employeur: Le débat Sur La connaissance du danger. Santé Mentale Et Droit.

[R73] Sayer A (2016). Why we can’t afford the rich.

[R74] Sennett R (1998). The corrosion of character: The personal consequences of work in the new capitalism.

[R75] Song Z, Baicker K (2019). Effect of a workplace wellness program on employee health and economic outcomes: A randomized clinical trial. Journal of the American Medical Association.

[R76] Stanley IH, Hom MA, Joiner TE (2016). A systematic review of suicidal thoughts and behaviors among police officers, firefighters, EMTs, and paramedics. Clinical Psychology Review.

[R77] Stone KW (2006). Flexibilization, globalization and privatization: Three challenges to labour rights in our time. Osgood Hall Law Journal.

[R78] Teoh K (2023). The value of occupational health and human resources in supporting mental health and wellbeing in the workplace. The Society of Occupational Medicine.

[R79] Tiesman HM, Konda S, Hartley D, Chaumont Menéndez C, Ridenour M, Hendricks S (2015). Suicide in U.S. workplaces, 2003–2010: A comparison with non-workplace suicides. American Journal of Preventive Medicine.

[R80] Tombs S (2016). Social protection after the crisis: Regulation without enforcement.

[R81] Tombs S, Whyte D (2007). Safety crimes.

[R82] Tombs S, Whyte D (2015). The corporate criminal: Why corporations must be abolished.

[R83] Waters S (2020). Suicide voices.

[R84] Waters S, Palmer H (2021). School of Languages, Cultures and Societies.

[R85] Waters S, Palmer H (2022). Dying at work. Work-related suicide – how does the UK regulatory context measure up?. Journal of Public Mental Health.

[R86] Waters S, Karanikolos M, McKee M (2016). When work kills. Journal of Public Mental Health.

[R87] Webb D (2010). Thinking about suicide: Contemplating and comprehending.

[R88] WHEC Workplace Health Expert Committee (2022). Work-related suicide WHEC-18 report.

[R89] White J (2017). What can critical suicidology do?. Death Studies.

[R90] White J, Marsh I, Kral MJ, Morris J (2016). Critical Suicidology: Transforming Suicide Research and Prevention for the 21st Century.

[R91] Whyte D, Wiegratz J, Whyte D, Wiegratz J (2016). Neoliberalism and the moral economy of fraud.

[R92] Windsor-Shellard B, Gunnell D (2019). Occupation-specific suicide risk in England: 2011–2015. British Journal of Psychiatry.

[R93] WorkSafe (2024). Work-related suicide. Examining the role of work factors in suicide.

